# Astragalus saponins modulates colon cancer development by regulating calpain-mediated glucose-regulated protein expression

**DOI:** 10.1186/1472-6882-14-401

**Published:** 2014-10-15

**Authors:** Yue Wang, Kathy K Auyeung, Xiaoyu Zhang, Joshua K Ko

**Affiliations:** Center for Cancer and Inflammation Research, School of Chinese Medicine, Hong Kong Baptist University, 7 Baptist University Road, Kowloon Tong, Hong Kong, China

**Keywords:** GRP78, Calpain inhibitor, ER stress, Gene silencing, AST, Apoptosis, Metastasis, Tumor xenograft

## Abstract

**Background:**

Glucose-regulated proteins (GRP) are induced in the cancer microenvironment to promote tumor survival, metastasis and drug resistance. AST was obtained from the medicinal plant *Astragalus membranaceus*, which possesses anti-tumor and pro-apoptotic properties in colon cancer cells and tumor xenograft. The present study aimed to investigate the involvement of GRP in endoplasmic reticulum (ER) stress-mediated apoptosis during colon cancer development, with focus on the correlation between AST-evoked regulation of GRP and calpain activation.

**Methods:**

The effects of AST on GRP and apoptotic activity were assessed in HCT 116 human colon adenocarcinoma cells. Calpain activity was examined by using a fluorescence assay kit. Immunofluorescence staining and immunoprecipitation were employed to determine the localization and association between calpains and GRP. GRP78 gene silencing was performed to confirm the importance of GRP in anticancer drug activities. The modulation of GRP and calpains was also studied in nude mice xenograft.

**Results:**

ER stress-mediated apoptosis was induced by AST, as shown by elevation in both spliced XBP-1 and CHOP levels, with parallel up-regulation of GRP. The expression of XBP-1 and CHOP continued to increase after the peak level of GRP was attained at 24 h. Nevertheless, the initial increase in calpain activity as well as calpain I and II protein level was gradually declined at later stage of drug treatment. Besides, the induction of GRP was partly reversed by calpain inhibitors, with concurrent promotion of AST-mediated apoptosis. The knockdown of GRP78 by gene silencing resulted in higher sensitivity of colon cancer cells to AST-induced apoptosis and reduction of colony formation. The association between calpains and GRP78 had been confirmed by immunofluorescence staining and immunoprecipitation. Modulation of GRP and calpains by AST was similarly demonstrated in nude mice xenograft, leading to significant inhibition of tumor growth.

**Conclusions:**

Our findings exemplify that calpains, in particular calpain II, play a permissive role in the modulation of GRP78 and consequent regulation of ER stress-induced apoptosis. Combination of calpain inhibitors and AST could exhibit a more pronounced pro-apoptotic effect. These results help to envisage a new therapeutic approach in colon cancer by targeting calpain and GRP.

**Electronic supplementary material:**

The online version of this article (doi:10.1186/1472-6882-14-401) contains supplementary material, which is available to authorized users.

## Background

Endoplasmic reticulum (ER) is the place for calcium storage and transmembrane protein synthesis, folding and maturation [1]. Apoptosis can be triggered by ER stress due to accumulation of misfolded proteins in the ER. On the one hand, prolonged or severe ER stress promotes several pro-apoptotic factors that result in apoptosis, while on the other hand it also activates a set of signaling pathway called unfolded protein response (UPR) to prevent apoptosis [2]. Transduction signals including CCAAT/enhancer-binding protein-homologous protein (CHOP), pro-apoptotic Bcl-2 family members, caspase 12 and c-Jun N-terminal kinase (JNK) all involve in ER stress-induced cell death [3], while UPR leads to adaptation for survival [4]. One of the protective mechanisms of body cells to adapt to ER stress is induction of the glucose-regulated protein (GRP) family members through glucose starvation. GRP78, also referred to as BiP (immunoglobulin heavy-chain binding protein), resides primarily in the ER, binds to calcium and serves as an ER stress signaling regulator [5]. Alternatively, GRP94 is the most abundant glycoprotein in the ER in vertebrates. Both GRP78 and GRP94 function as molecular chaperones that can bind to misfolded proteins and unassembled the complexes to protect cells against death [5, 6]. The anti-apoptotic property of GRP could lead to cancer progression and drug resistance in neoplastic cells [7, 8]. Another study also showed that GRP may promote tumor cell proliferation and metastasis, which have major implications in the prognosis and treatment of cancer [9]. Down-regulation of GRP78 in colorectal cancer cells facilitates the sensitization to paclitaxel-induced apoptosis [10]. Several natural compounds including genistein and green tea constituents have been proven to strengthen the anticancer ability through GRP78 inhibition [11]. These observations have provided evidence that inhibitors of GRP78 can be used in combination with standard chemotherapeutic agents to enhance drug efficacy against cancer development.

Calcium has been known to be crucial in the regulation of apoptotic signaling [12]. Elevation of intracellular calcium level activates several pro-apoptotic proteins including phosphatase calcineurin and a family of calcium-dependent cysteine proteases called calpains [13]. Calpains can be found in all eukaryotes with two ubiquitous isoforms, *μ*-calpain and *m*-calpain (also known as calpains I and II, respectively). Metastasized tumors from human renal cell carcinoma were found to have overexpression of calpain I [14], whereas epigenetic activation of calpain II could be essential in determining the invasiveness of human prostate cancer cells [15]. Release of calcium from the ER would lead to calpain activation. Calcium-activated calpains are translocated from the cytosol to the cell membrane, where they associate with caspases to regulate apoptosis. These activated calpains trigger degradation of the plasma membrane and subsequently result in the loss of cellular integrity [16]. Calpains are involved in the execution phase of apoptosis when induced by certain stimuli [17]. The actual role of calpain can vary depending on the type and severity of the apoptotic stimulus even within the same cell line [18, 19]. In general, calcium-activated calpains are translocated from the cytosol to the cell membrane, where they crosstalk with caspases to regulate apoptosis. Based on the available evidence, apoptotic regulation by calpains can be both positive and negative [20]. It was reported that calpain II can cleave procaspase 12 to generate active caspase 12, which is capable of directly activating caspase 3 that leads to apoptosis [21]. The role of calpains in apoptosis is also indicated by a growing list of its substrates, including p53, poly(ADP-ribose) polymerase (PARP), Bax, Bid, apoptosis-inducing factor (AIF) and several cytoskeletal proteins [22]. Recent research has shown that inhibition of calpain activity and Bax deficiency would promote the enhancement of autophagy in hepatocellular carcinoma. It has been suggested that calpain could act as a molecular link between autophagy and apoptosis that reveals a possible crosstalk between the two closely related processes [23]. Other than promotion of cell death, calpains also participate in the regulation of adhesion complexes that are important in cell migration and motility [24]. Based on the above observations, calpains have already been regarded as a potential new target for anti-cancer drugs.

It had been recently demonstrated in our laboratory that AST, the total saponins being isolated from the root of the medicinal herb *Astragalus membranaceus* possess anti-tumorigenic activity in several cancer cell types including hepatocellular carcinoma (HCC), colon cancer cells and xenografts [25–27]. AST facilitates growth inhibition and promotes apoptosis in HepG2 cells through modulation of an ERK-independent NF-κB signaling pathway [25]. AST could also induce extrinsic apoptotic cascade and cause cell cycle arrest in HT-29 cells by modulation of both mTOR and ERK signaling, with concomitant inhibition of NF-κB in the latter mechanism [28]. Last but not least, we have revealed that non-steroidal anti-inflammatory drug activated gene (NAG-1) is a potential molecular target of AST in its anti-tumorigenic and pro-apoptotic actions [26]. The aim of the present study was to explore whether AST is able to suppress GRP and regulate calpains in human colon cancer cells, which could contribute to its anti-tumor property. Close interactions between GRP and calpains would promote apoptosis of cancer cells. Hence, a new and effective therapeutic intervention of colon cancer growth with known molecular targets could be established in the coming future.

## Methods

### Materials

Dulbecco’s modified essential medium, fetal bovine serum (FBS), penicillin and streptomycin were supplied by Gibco (Carlsbad, CA). The enhanced chemiluminescence (ECL) detection kit was purchased from Amersham Biosciences (Piscataway, NJ). 3-[4, 5-dimethylthiazol-2-yl) 2,5-diphenyltetrazolium bromide (MTT) and other chemicals were obtained from Sigma-Aldrich (St. Louis, MO) unless specified. The following antibodies were used: anti-GRP78, anti-GRP94, anti-calpain I and anti-calpain II (Santa Cruz Biotechnology, Santa Cruz, CA); anti-caspase 3, (Upstate Biotechnology, Charlottesville, VA); anti-PARP (BD Pharmingen, San Jose, CA); anti-β-actin (Sigma-Aldrich, St. Louis, MO).

### Cell culture

Human colorectal carcinoma cells HCT116 were obtained from the American Type Culture Collection (ATCC; Manassas, VA). Cells were grown in 75-cm^2^ flasks and incubated at 37°C under 5% CO_2_ atmosphere in Dulbecco’s Modified Eagle’s medium (DMEM) supplemented with 10% FBS plus 1% penicillin and streptomycin (Invitrogen, Carlsbad, CA).

### Preparation of AST

Radix *Astragalus membranaceus* (Fisch.) Bunge var. *mongolicus* was obtained from the province of Shanxi, China through the MMCHY Chinese Medicine Specialty Clinic and Good Clinical Practice Centre of our institution. Microscopic and chromatographic analyses as well as DNA fingerprinting were carried out to determine the authenticity and to assess the quality of raw herb in the Quality Assurance Laboratory of the School of Chinese Medicine, Hong Kong Baptist University. To ensure consistency between batches, voucher specimens will be kept at the herbarium centre for future reference. AST was extracted according to the method as described previously. In brief, 500 g of crude herb was refluxed in methanol for 1 h. n-Butanol was then added to the re-constituted residue for phase separation to obtain the total Astragalus saponins [29]. Butanol was removed in the rotary evaporator. The resulting residue was reconstituted with distilled water and lyophilized into dry powder.

### Assessment of cell viability by MTT assay

Upon drug treatment, the cell viability assay was employed to determine the effective concentration of AST. Cell viability of HCT116 was measured by using the 3-(4,5-dimethylthiazol- 2-yl)-2,5-diphenyltetrazolium bromide (MTT) reduction method, which involved the reduction of yellow MTT into purple insoluble formazan product by the mitochondrial reductase enzymes of viable cells. HCT116 cells (3.0 × 10^3^ cells) were plated in 96 well plates in DMEM and treated with AST for 24, 48 or 72 h, followed by incubation with MTT (5 mg/ml) for another 3 h at 37°C. Control cells received DMEM treatment only. DMSO was then used to dissolve the formazan product for spectrophotometric analysis at the wavelength of 540 nm.

### Western immunoblotting

Protein levels after drug treatments were evaluated using Western immunoblotting. Cells were seeded at a density of 3.5 × 10^5^ in 60-mm dishes. After drug treatment at various time points, cells were lysed in RIPA buffer (pH 7.4) containing 50 mM Tris, 150 mM NaCl, 0.5% deoxycholate, 0.1% SDS, 2 mM EDTA, 0.1% Triton X-100, 10% glycerol, 1 mM phenylmethylsulfonyl fluoride and 10 μg/ml aprotinin. After centrifugation at 14,000 × *g* for 10 min at 4°C, the insoluble materials were removed and proteins were quantified using Coomassie Plus Protein Assay Reagent kit (Pierce, Rockford, IL). Total cellular proteins (20–40 μg) in the cell lysate were separated by 8-15% SDS polyacrylamide gel electrophoresis and transferred onto a nitrocellulose membrane. Protein bands were detected by incubating the membrane in respective primary antibodies (1:1000) and secondary antibodies (1:2000) conjugated with horseradish peroxidase, and visualized by adding enhanced chemiluminescence (ECL) reagents (Amersham Biosciences; Piscataway, NJ). Results were analyzed by using the Quantity One version 4.4.1 Basic software (BioRad, Hercules, CA).

### Immunofluorescence staining

The immunofluorescence staining was used to examine the co-localization of GRP78 and calpain II. HCT116 cells were grown on glass coverslips treated with AST for 48 h. After treatment, cells were washed with cold phosphate buffered saline (PBS) twice and fixed in 4% paraformaldehyde for 10 min. Following fixation, cells were washed with PBS for three times and then permeabilized with ice-cold methanol at −20°C for 5 min. Cells were then washed with PBS for three more times before incubating with primary antibodies (1:100 dilution) at 4°C. On the next day, the slides were stained with corresponding FITC-conjugated secondary antibodies for 2 h at room temperature. The nuclei were then stained with the DNA binding dye 4′,6-diamidino-2-phenylindole dihydrochloride (DAPI) for 10 min and examined under fluorescence microscopy (Leica DMI3000B, Leica Microsystems, Wetzlar, Germany).

### Immunoprecipitation

The association and dynamic interaction between GRPs and calpains were studied using immunoprecipitation. After drug treatment, HCT116 cells were harvested and lysed in a immunoprecipitation (IP) buffer containing 50 mM Tris-Cl, pH 7.5, 150 mM NaCl, 1 mM EDTA, 1 mM EGTA, 0.1% NP-40, 1 mM phenylmethylsulfonyl fluoride and 10 μg/ml aprotinin. Supernatant was collected after centrifugation at 14,000 × *g* at 4°C. The protein G sepharose resins were pre-cleared and restored to 50% slurry with IP buffer. Antibody (1 μg) was then added to each prepared sample and rocked overnight at 4°C. The immunocomplex was captured by adding pre-cleared protein G sepharose beads slurry. After incubation at 4°C for 1 h, the sepharose resins were collected by pulse centrifugation at 4°C (10 s at 14,000 × *g*) and washed twice with ice-cold PBS. Proteins were boiled in 5× sample loading buffer for 5 min to dissociate the immunocomplexes from the sepharose resins, and stored at −20°C for subsequent use.

### siRNA transfection

We performed the siRNA transfection so as to confirm the involvement of GRP78 in AST-mediated. Cells were transfected with GRP78 siRNA duplexes containing a pool of 3 specific 19–25 nt siRNAs targeting GRP78 (Santa Cruz Biotechnology). A non-targeting 20–25 nt scramble siRNA (Scr) was used as negative control. In brief, control (medium-treated) or AST-treated (80 μg/ml) HCT116 cells were cultured at 60% confluence and transfected with GRP78 siRNA or Scr for 24 h using Lipofectamine (Invitrogen) according to the manufacturer’s instruction. Following gene silencing, Western immunoblotting was conducted to assess target proteins in cells.

### Colony formation assays

Colony formation assay was based on the ability of a single cell to grow into a colony and was used to study the effectiveness of specific agents on the survival and proliferation of cells. After gene silencing of GRP78, cells were seeded in six-well plates at the density of 200 cells/well and cultured in the absence or presence of AST for 14 days. Medium was changed every 4 days to maintain the viability of cells and the propagation to form visible colonies. The colonies were fixed with methanol-acetic acid (3:1), and stained with 1% crystal violet for 2 h at room temperature. The number of colonies in each well was counted.

### Calpain activity assay

Calpain Activity Assay kit involved fluorometric detection of protease calpain activity in the cell, in which only activated calpain in cytosol was detected (Abcam, Cambridge, MA). In brief, HCT116 cells (1 × 10^6^) were suspended in 100 μl of extraction buffer and centrifuged at 10,000 × *g* for 1 min. Cell lysate (~200 μg) was diluted in 85 μl of extraction buffer. For the positive control, 2 μl of active calpain was added to 85 μl of extraction buffer. The untreated cell lysate was used as negative control. After incubation at 37°C for 1 h, the absorbance of samples were read in a fluorescence spectrometer equipped with a 400 nm excitation filter and a 505 nm emission filter (PerkinElmer LS55, Waltham, MA).

### Tumor xenograft in nude mice

The involvement of caplain and GRP in AST-mediated modulation of colon cancer development was studied using tumor xenograft. All the experimental procedures involving laboratory animals in this original research were approved by the Committee on the Use of Human & Animal Subjects in Teaching & Research (HASC) of our home institution, and had been performed according to international, national and institutional regulations. Cell suspension was obtained by trypsinization of confluent HCT116 cells. Balb/c-*nu*/*nu* mice were randomly assigned into control (vehicle) and AST (100 mg/kg, p.o. once daily for 14 days) treatment groups (n = 6-8). Animals were anesthetized and cell suspension (2 × 10^6^ in 100 μl medium) was injected subcutaneously near the right flank of each animal. Tumors became palpable 10 days after implantation, when vehicle or drug treatment was commenced. Tumor volume was measured every other day by using a digital caliper and calculated as (length × width^2^)/2. The percentage of change in tumor volume with respect to the measured volume on day 10 (100%) was determined until the end of the experimental period. Body weight as well as the amount of food and water intake in all animals was monitored as assessment of possible drug toxicity. Any mortality during the course of study would also be noted. The mass of the tumor in each animal was assessed at the end of experiment on day 24. Protein levels of GRP78, GRP94, calpain I and calpain II were determined in the excised tumor samples by using Western immunoblotting.

### Statistical analysis

Results were expressed as mean ± SEM. Statistical significance was determined by either one- or two-way analysis of variance (ANOVA) followed by Tukey post-hoc test (SPSS version 10.0, Chicago, IL), whereas *p* <0.05 was deemed to be significant.

## Results

### AST induced ER stress-mediated apoptosis

**Figure 1:**
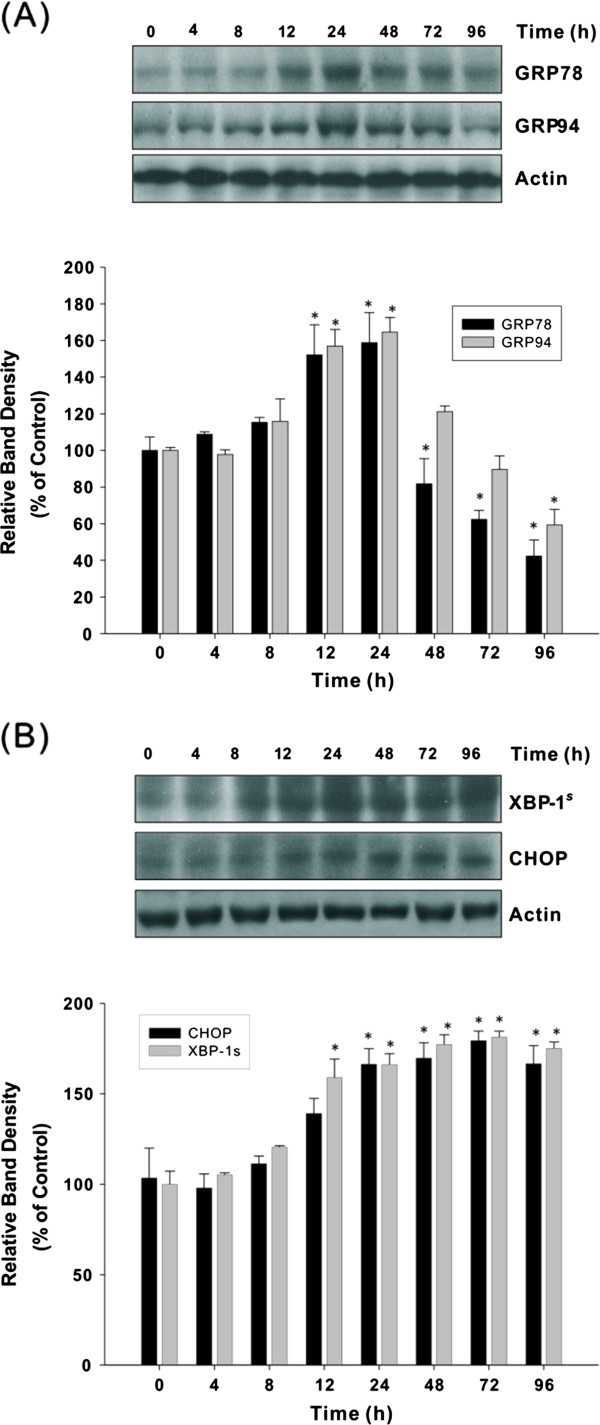
**Effects of AST on UPR activation (A) and ER stress-induced apoptosis (B) on HCT116 colon cancer cells.** HCT116 colon cancer cells were treated with AST (80 μg/ml) for different time intervals (4–96 h). Specific antibodies against GRP78, GRP94, XBP-1^s^ and CHOP were used in Western immunoblotting to detect the expression of different proteins. Data shown are representative immunoblots with similar findings, normalized by β-actin. Blots were scanned and optical densities were determined using the Quantity One software. Each histogram represents mean ± SEM of four independent experiments. *p <0.05 compared with the protein level at zero time.

In order to explore the involvement of ER stress and UPR activation in AST-induced apoptosis, the drug effects on GRP78 and GRP94 (the protective arm of the UPR), CHOP (also known as the growth arrest- and DNA damage-inducible gene GADD153) as well as splicing of XBP-1 (the pro-apoptotic component of ER stress) were examined. Figure 1A illustrates that AST elicited an ER stress response, as shown by the enhanced expression of both GRP78 and GRP94 at the early time points. Maximum GRP expression was attained at 24 h and began to decline gradually, suggesting that prolonged ER stress caused failure in the ER recovery process, leading to the initiation of apoptotic cell death. Upon continuous AST treatment, the expression of CHOP and spliced XBP-1 kept increasing even after the peak of GRP expression were reached (Figure 1B). These results exemplify that at early stage of AST treatment, the response of colon cancer cells to ER stress was initially cytoprotective. Nonetheless, upon prolonged drug treatment when cells were challenged by overwhelming amount of ER stress, apoptosis would be induced, along with concomitant down-regulation of GRP.

### Involvement of calpains in AST-induced ER stress-mediated apoptosis

**Figure 2:**
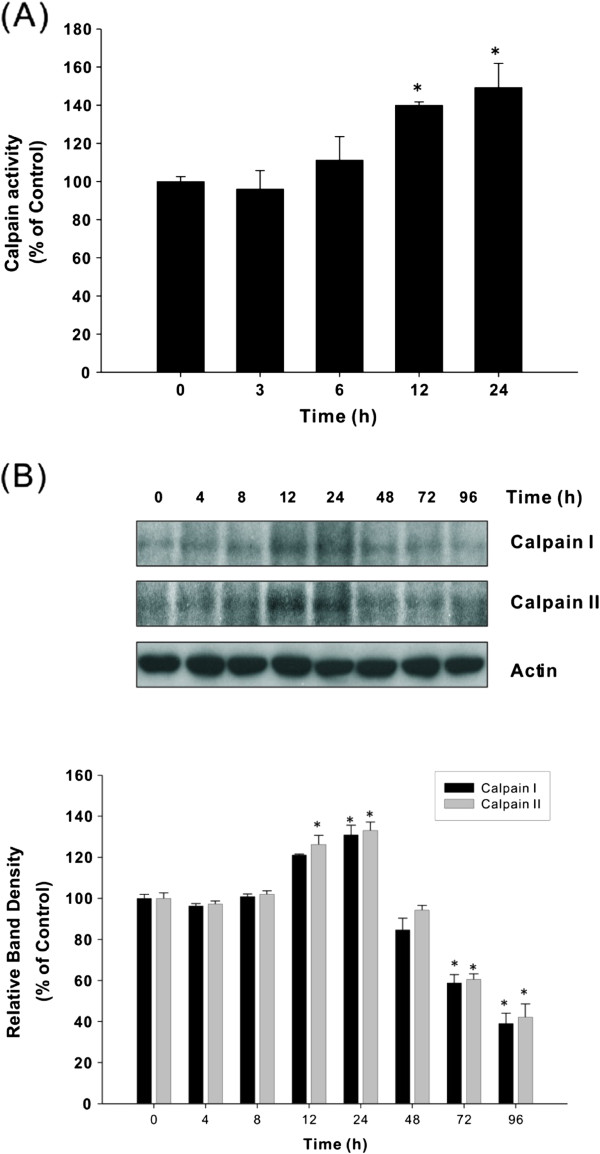
**AST modulates calpain activity (A) and calpain protein expression on HCT116 colon cancer cells (B).** HCT116 colon cancer cells were treated with AST (80 μg/ml) for different time intervals (4–96 h). Calpain activity was measured by employing the calpain activity assay using fluorescent calpain substrate Ac-LLY-AFC. Calpain I and II protein levels in AST-treated cells were assessed by Western immunoblotting using specific antibodies. Data shown are representative immunoblots with similar findings, normalized by β-actin. Blots were scanned and optical densities were determined using the Quantity One software. Each histogram represents mean ± SEM of four independent experiments. *p <0.05 compared with the protein level at zero time.

We further investigated the signaling pathway of ER stress-mediated apoptosis by studying the effect of AST on the activation of calpains, the indicator of calcium homeostasis disruption. Calpain activity (Figure 2A) as well as calpain I and II protein expression (Figure 2B) were elevated following AST treatment. The induced expression of calpain proteins was gradually declined at later stage of drug treatment after 24 h. These findings may be explained by some reports stating that lack of calpain activity could sensitize cells to programmed cell death due to a variety of stimuli, including growth factors and nutrient starvation [30].

**Figure 3:**
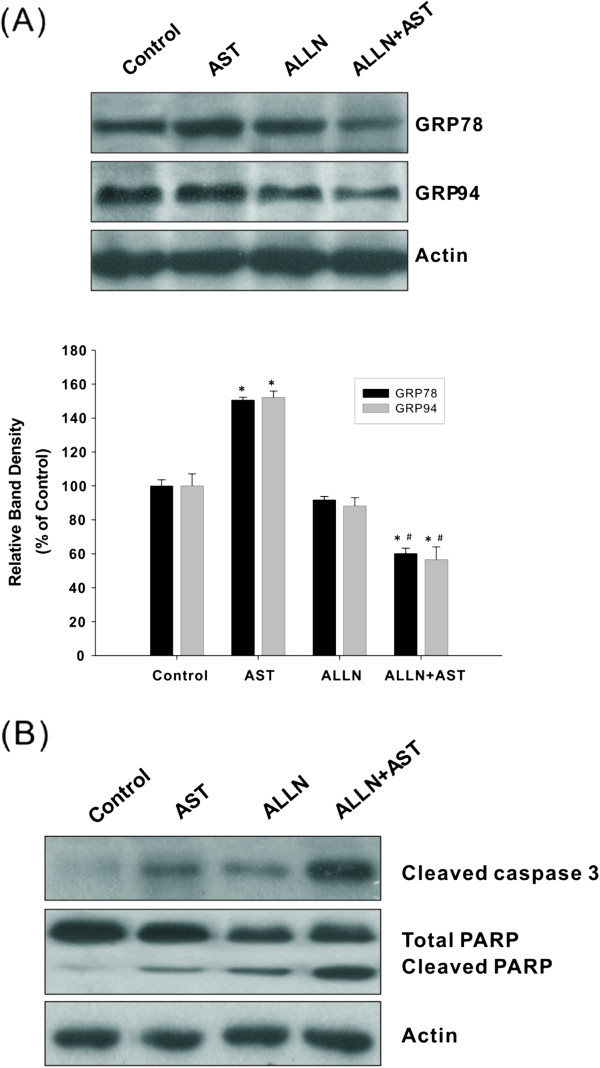
**Calpain inhibitor attenuates GRP induction (A) and promotes AST-induced apoptosis (B).** HCT116 colon cancer cells were either incubated with culture medium only (control) or pre-treated with the calpain inhibitor ALLN for 30 min. AST (80 μg/ml) was then administrated to cells and incubated for 24 h in all groups except the ALLN alone group. Specific antibodies against GRP78, GRP94, cleaved caspase-3 and PARP were used in Western immunoblotting to detect the expression of different proteins. Data shown are representative immunoblots with similar findings, normalized by β-actin. Blots were scanned and optical densities were determined using the Quantity One software. Each histogram represents mean ± SEM of four independent experiments. *p <0.05 compared with the control without drug treatment; ^#^ *p* <0.05 compared with cells treated with ALLN alone.

In order to examine the role of calpains in AST-induced apoptosis, regulation of GRP was assessed in the presence of calpain inhibitors. As shown in Figure 3A, the induction of GRP78 and GRP94 by AST was significantly suppressed by the calpain inhibitor ALLN, implicating the enhancement of ER stress-associated apoptosis. This was confirmed by the observation of increased PARP cleavage and caspase-3 activation (Figure 3B). Similar results were obtained by using another calpain inhibitor ALLM (data not shown). We have shown here that calpain inhibitors could potentiate the pro-apoptotic effect of AST through down-regulation of GRP, leading to augmentation of anti-cancer drug activity. These findings provide evidence on the fact that sensitization of colon cancer cells to ER stress-induced apoptosis by inhibition of calpain is associated with GRP modulation.

### Co-localization and dynamic interaction between calpain II and GRP78

**Figure 4:**
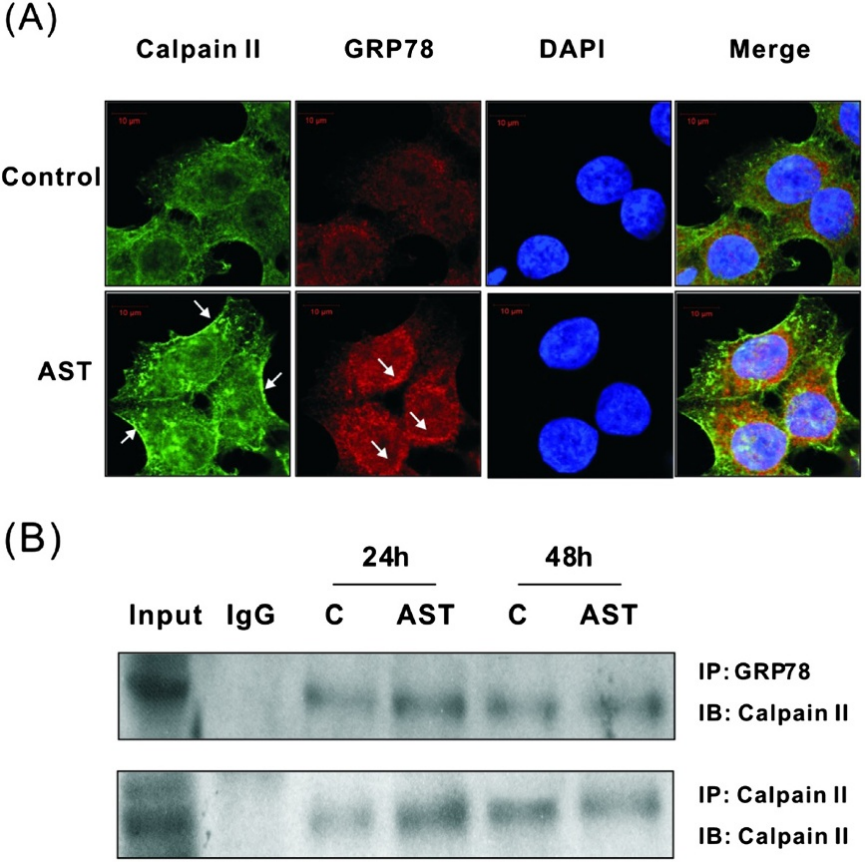
**Effects of AST on the localization of (A) and interaction between calpain II and GRP78 (B) were determined.** HCT116 cells were treated with AST (80 μg/ml) for 24 h. Co-localization of calpain II and GRP78 were detected in control [C] and drug-treated cells. Specific immunofluorescence antibodies against calpain II and GRP78 were used, while DAPI acted as nuclear stain. Green and red fluorescence indicates the presence of calpain and GRP78, respectively in the cells and blue color represents the DAPI-stained nucleus. Interaction between calpain II and GRP78 was further detected by immunoprecipitation [IP]. HCT116 cells were treated with AST (80 μg/ml) for 24 or 48 h. Immunoprecipitated proteins were collected and subject to Western immunoblotting [IB]. Data shown are representative immunoblots with similar findings. IgG was used as precipitating antibody in the negative control.

In order to elucidate the regulatory role of calpain on GRP during ER stress-induced apoptosis, immunofluorescence staining was performed. Under our experimental condition, a significant association was observed between calpain II and GRP78 after 24 h of drug treatment (Figure 4A). There was only a minimal amount of calpain II signal being detected in the cytoplasm and nucleus in untreated control cells, whereas a marked increase of expression could be observed in the cell membrane of drug-treated cells. Likewise, the expression of GRP78 was relatively weak in untreated control cells when compared with the induction of cell membrane expression after drug treatment. The enhancement of signal in the cell membrane indicates that AST-induced ER stress could facilitate GRP78 localization on the cell surface. The interaction between GRP78 and calpain II had been indicated as shown by the increased co-localization after drug treatment, especially on the cell membrane. In order to confirm the results from immunofluorescence staining, immunoprecipitation was performed, followed by Western immunoblotting. Figure 4B demonstrates that endogenous GRP78 was co-immunoprecipitated with calpain II. The interaction between endogenous GRP78 and calpain II should be specific because this complex was not observed when using control IgG as the precipitating antibody. The amount of GRP78 that was co-precipitated with calpain II increased after 24 h of drug treatment. This observation is coherent with the increased amount of activated calpain II in the precipitates, presumably due to increased cellular contents of the calpains. The findings from immunofluoresence staining and immunoprecipitation have revealed a dynamic interaction between GRP78 and calpain II in human colon cancer cells following AST treatment.

### Suppression of GRP78 by small interfering RNA enhances sensitivity of HCT116 colon cancer cells

**Figure 5:**
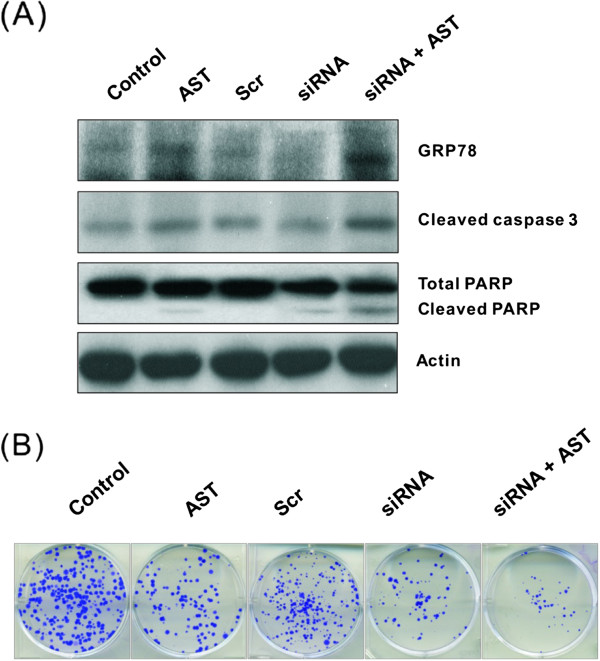
**Effects of GRP78 silencing on AST-induced apoptosis (A) and colony formation (B).** After 24 h of transfection with GRP78 siRNA or scrambled control siRNA [Scr], the protein levels of GRP78, cleaved caspase 3 and PARP were determined in HCT116 cells with or without AST treatment (80 μg/ml for 24 h) by Western immunoblotting. Data shown are representative immunoblots from four independent experiments with similar findings, normalized by β-actin. Blots were scanned and optical densities were determined using the Quantity One software. Alternatively, native or GRP78 gene-silenced HCT116 cells were cultured in six-well plates at the density of 200 cells/well at 37°C in 5% CO_2_ atmosphere for 3 weeks with or without AST treatment (80 μg/ml) in the last 14 days to determine colony formation.

To further validate the role of GRP78 in ER stress-induced apoptosis, the gene expression of GRP78 was silenced. Figure 5A exemplifies a significant knock-down of GRP78 (by more than 70%) after 24 h of siRNA transfection, without alteration of apoptosis (indicated by the unchanged level of cleaved caspase-3 and PARP cleavage). Nonetheless, GRP78 expression had been increased by more than 6 folds following AST treatment in GRP78-silenced cells, with concurrent increase in apoptosis (with increased PARP cleavage and caspase-3 activation). These results demonstrate that GRP78 knock-down by gene silencing would result in a substantial increase in the sensitivity of colon cancer cells to apoptosis. The effect of GRP78 gene silencing on colony formation was further assessed (Figure 5B). Cell colony formation (according to both number and size) was significantly reduced in AST-treated GRP78-silenced cells when compared to either control GRP78-silenced cells or AST-treated cells without gene silencing.

### Effects of AST on GRP, calpains and tumor growth in HCT116 xenografted nude mice

**Figure 6:**
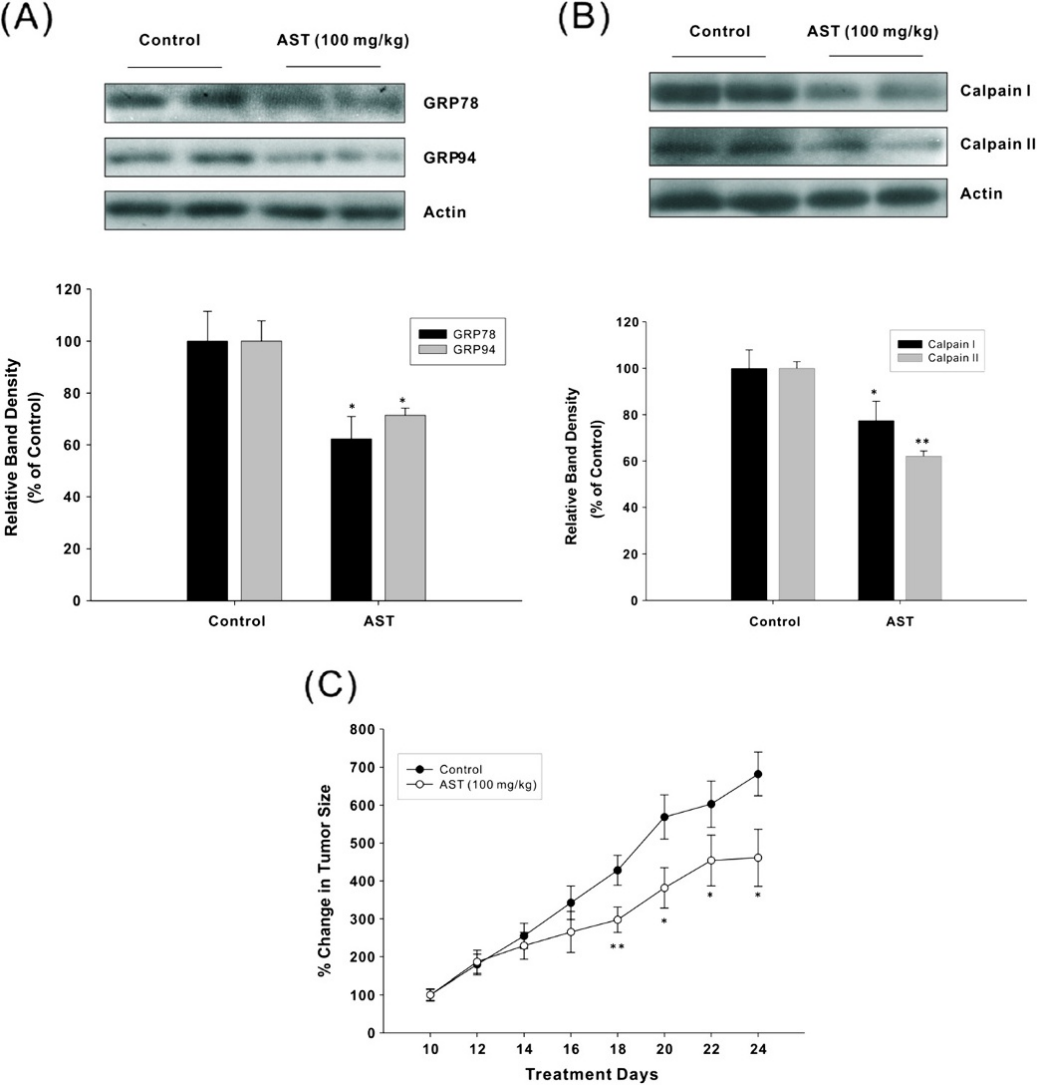
**AST modulates the protein expression of GRP (A) and calpains (B) in tumor tissues, and inhibits tumor growth in HCT116-xenografted nude mice (C).** HCT116 tumor cells (2 × 10^6^) were injected subcutaneously to Balb/c-*nu/nu* mice near the right flank on day 0. Drug treatment (AST: 100 mg/kg) commenced from day 10 when tumors became palpable, and the tumor growth in xenografted animals were monitored every other day. Animals in each treatment group (n = 6-8) were sacrificed on day 24 of the experiment, when the tumor tissues were excised to assess the protein expression of GRP78, GRP94, calpain I and calpain II by using Western immunoblotting. Percentage change in tumor volume in each treatment group from day 10 to day 24 was measured and compared with respect to the volume on day 10 (100%). Representative immunoblots were obtained from independent experiments with similar findings, normalized by β-actin. Blots were scanned and optical densities were determined using the Quantity One software. The histogram represents mean ± SEM of four independent experiments. *p <0.05 and **p <0.01 compared with untreated control mice.

In order to confirm that AST could modulate tumor development by regulating calpain-mediated GRP level, the expression of GRP and calpains was assessed in HCT116-xenografted nude mice. Results show that AST treatment markedly reduced the expression of GRP78, GRP94 and calpain II in colonic tumor tissues, with less significant downregulation of calpain I (Figure 6A and B). Besides, there was also a significant inhibition of tumor growth from day 18 to day 24 of the experimental period (Figure 6C), which together verifies our *in vitro* data.

## Discussion

ER has been designated as a major therapeutic drug target due to its vital involvement in apoptotic execution [31]. Perturbation of ER homeostasis plays critical roles in tumorigenesis, and therapeutic modulation of ER chaperones and/or UPR components presents potential anti-tumor treatment. Nevertheless, excessive activation of the ER stress pathways in hypoxic tumor cells has been shown to render them more sensitive to proteasome inhibitors, resulting in increased cytotoxicity [32]. In the present study, AST has been proven to facilitate ER stress-induced apoptosis through regulation of GRP78. It fact, most of the GRP in tumor cells are engaged in the formation of multi-chaperone complexes, an event not being observed in normal cells [33]. Evidence has shown that high levels of GRP78 and GRP94 were detected in breast and colon cancer [6]. Cell surface GRP78 has been identified as an important tumor cell signaling and viability regulator by forming complexes with a variety of cell-surface proteins to regulate proliferation and cell viability [34, 35]. Intracellular GRP78 possesses pro-survival and anti-apoptotic functions while the cell surface GRP78 serves as a receptor for pro-apoptotic ligands such as Kringle (K5) and Par-4, promoting apoptosis [36, 37]. In addition, it is known that prolonged ER stress may activate specific mechanisms for GRP78 to aggressively interact with other pro-apoptotic proteins such as caspase 7 and T-cadherin [38, 39]. In the present study, AST has been proven to facilitate ER stress-induced apoptosis through regulation of GRP78. An early increase of GRP78 level plays an essential role in the protection of cells from apoptosis during ER stress [37, 38]. In particular, a marked increase of GRP78 expression in the cell membrane of drug-treated cells could be observed, while gradual decrease of GRP78 in prolonged ER stress caused failure in the ER recovery process and led to the initiation of apoptotic cell death. Until now, the mechanism of GRP78 trafficking from the ER to the cell surface is not well understood. Nevertheless, it is plausible that ER luminal GRP78, either by itself or through interaction with other proteins being translocated into the ER, could be redistributed to the cell surface by escaping the ER retrieval mechanism [40]. In addition, GRP regulation may require a close working relationship with the tumor suppressor gene *p53* to promote apoptosis under different tumor microenvironments [41]. Mutation of *p53* could however affect both caspase and calpain functions in the control of apoptosis [42]. This could explain why AST-induced downregulation of GRP was largely attenuated in another colonic cancer cell line with mutated *p53* gene (Additional file 1: Figure S1). It is to our surprise that silencing of GRP78 alone did not impose a significant promotion of apoptosis in non-drug-treated HCT116 colon cancer cells. Nonetheless, suppression of GRP78 protein expression by siRNA deactivated UPR and potentiated AST-induced apoptosis in the cells. This has been further verified by using colony formation assay.

GRP78 is known to function as a Ca^2+^-binding protein in the ER thereby preventing Ca^2+^ efflux into the cytosol and providing a buffer against induced increases in cytosolic Ca^2+^ levels, which could result in cell death. On the other hand, increased cytosolic calcium will cause the activation of calpain and its membrane translocation, where they were associated with caspases, such as caspase-12, to regulate apoptosis [43]. Furthermore, it is known that GRP78 could form a complex with caspase-7 and −12 and prevents release of caspase-12 from the ER [44]. Here, we have unveiled a dynamic interaction between GRP78 and calpain II following treatment of pro-apoptotic agent. GRP78 is a physiological substrate for calpain II, which will be activated at the ER membrane by interacting with GRP78 upon drug stimulation. Further studies are required to prove whether GRP78 can directly or indirectly interact with calpain II through caspases. However, a similar study showed that GRP94 is a physiological substrate for calpain. Calpain has been demonstrated to be activated at the ER membrane, where it interacts with GRP94, resulting in its specific proteolytic cleavage as the cells undergo apoptosis [45]. Our results have indicated that the peak level of GRP78 was attained after 24 h of ER stress. This is in fact in line with a previous report that the most significant increase in caspase 3 activity was observed at 24 h of drug-induced ER stress, simultaneous to increased caspase 12 enzymatic activity [46]. They explained that caspase 12 was first initially processed by calpain by removing the CARD-containing pro-domain to produce a 38-kDa fragment. The above discovery implicates that sensitization of colon cancer cells to ER stress-induced apoptosis by regulation of calpain is associated with blockade of prolonged GRP78 induction. Calpain is implicated in anti-apoptotic functions *via* stimulation of ERK pathway in neurons [47] as well as NF-κB survival pathway following treatment of tumor necrosis factor [48]. Proteasome inhibitor PS-341 (bortezomib)-induced IκBα degradation was prevented by calpain inhibition, leading to an enhancement of the anti-cancer activity [49]. Silencing of calpain II resulted in a partial restoration of irinotecan sensitivity in drug-resistant colorectal cancer cells [50]. Therefore, we have determined that combination of pharmacological calpain inhibitor and other chemotherapeutic adjuvants could become a new therapeutic strategy for human colon cancer treatment through promotion of apoptosis. The induction of GRPs following exposure to AST was significantly suppressed by the calpain inhibitors. This part of result has suggested that calpain activation was an early event and may precede the disruption of the ER. It was possible that the calpain inhibitor interfered with the induction of GRPs and the subsequent apoptotic response. In addition, results from the current work have revealed that combination of AST and calpain inhibitors actually resulted in augmentation in the induction of ER stress-associated apoptosis. In spite of this, some transmembrane proteins that promote cancer cell migration, invasiveness and metastasis by provoking cell motility were found to be down-regulated by calpain-mediated proteolytic degradation [51]. Calpain II was overexpressed in colorectal cancer biopsy samples and played an important role in early stages of the metastatic process [52]. Elevated calpain activity is necessary for the remodeling of focal adhesion, and cell migration of oncoproteins, in which they play distinctive roles in oncogenic events induced by individual transforming proteins [53]. It was shown that inhibition of calpain activity could modulate cell migration through stabilized peripheral focal adhesions and by decreasing the rate of cell membrane detachment, all aiming to gain control of cancer cell metastasis [54]. Other than being self-driven, calpains could also interact with other signaling pathways in the regulation of essential cellular functions and diseases, including cancer. It was reported that calpain small subunit 1 interacts with class IA phosphoinositide 3-kinases (PI3K) to induce a negative regulation of the PI3K-Akt pathway [55]. The p110α, p110β and p85s subunits of PI3K are substrates of calpains I and II, with preferential cleavage of p110, which leads to inhibition of PI3K activity and suppressed downstream protein expression. This calpain-PI3K modulation plays an important role in the regulation of PI3K-Akt signaling, particularly in response to stress such as serum starvation. We have previously reported that AST inhibits colon cancer growth by its interaction with the PI3K-Akt-mTOR pathway [26, 28], which may correlate with the complicated modulation of calpains. AST can be used as an adjuvant in combination with other orthodox chemotherapeutic drugs to reduce the side effects of the latter compounds [27]. We have also demonstrated that AST could exert anti-carcinogenic effects against colon, liver and gastric cancers through different mechanisms [25, 28]. Besides, AST possesses anti-angiogenic and anti-invasive properties in colon [56] and gastric cancer cells [57].

## Conclusions

The present study exemplifies that calpain II play an essential role in the modulation of GRP78 and regulation of ER stress-induced apoptosis in colon cancer. The combination of AST with calpain inhibitors could exhibit a more pronounced effect on ER stress-associated apoptosis. These findings suggest a new therapeutic approach by targeting calpain and GRP in the treatment of human colon cancer.

## Electronic supplementary material

Additional file 1: Figure S1: AST modulates the protein expression of GRP in HT-29 colon cancer cells. HT-29 colon cancer cells were treated with AST (80 μg/ml) for different time intervals (12–72 h). Specific antibodies against GRP78 and GRP94 were used in Western immunoblotting to detect the expression of different proteins. Data shown are representative immunoblots with similar findings, normalized by β-actin. Blots were scanned and optical densities were determined using the Quantity One software. (TIFF 32 KB)
